# A Pluridisciplinary Tracheostomy Weaning Protocol for Brain-Injured Patients, Outside of the Intensive Care Unit and Without Instrumental Assessment: Results of Pilot Study

**DOI:** 10.1007/s00455-023-10641-7

**Published:** 2023-12-07

**Authors:** Thomas Gallice, Emmanuelle Cugy, Christine Germain, Clément Barthélemy, Julie Laimay, Julie Gaube, Mélanie Engelhardt, Olivier Branchard, Elodie Maloizel, Eric Frison, Patrick Dehail, Emmanuel Cuny

**Affiliations:** 1 Neurosurgery Unit B, Bordeaux University Hospital, Pellegrin Hospital, 33000 Bordeaux, France; 2 Bordeaux Research Center for Population Health (BPH), Team: ACTIVE, University Bordeaux Segalen, UMR_S 1219, 33000 Bordeaux, France; 3 Physical and Rehabilitation Medicine Unit, Swallowing Evaluation Unit, Bordeaux University Hospital, Tastet-Girard Hospital, 33000 Bordeaux, France; 4 Neurological ICU, Bordeaux University Hospital, Pellegrin Hospital, 33000 Bordeaux, France; 5 Arcachon Hospital, Physical and Rehabilitation Medicine Unit, 33260 La Teste de Buch, France; 6 Medical Information Unit, Bordeaux University Hospital, Pellegrin Hospital, 33000 Bordeaux, France; 7 Physical and Rehabilitation Medicine Unit, Bordeaux University Hospital, Tastet-Girard Hospital, 33000 Bordeaux, France; 8 Physical and Rehabilitation Medicine Unit, Cognition and Language Unit, Bordeaux University Hospital, Tastet-Girard Hospital, 33000 Bordeaux, France; 9 Neuro-Vascular Unit, Bordeaux University Hospital, Pellegrin Hospital, 33000 Bordeaux, France; 10 Neurodegenerative Diseases Institute, CNRS, UMR 5293, 33000 Bordeaux, France

**Keywords:** Acquired brain injury, Post intensive care unit, Rehabilitation, Tracheostomy weaning, Decannulation

## Abstract

Concurrently to the recent development of percutaneous tracheostomy techniques in the intensive care unit (ICU), the amount of tracheostomized brain-injured patients has increased. Despites its advantages, tracheostomy may represent an obstacle to their orientation towards conventional hospitalization or rehabilitation services. To date, there is no recommendation for tracheostomy weaning outside of the ICU. We created a pluridisciplinary tracheostomy weaning protocol relying on standardized criteria but adapted to each patient’s characteristics and that does not require instrumental assessment. It was tested in a prospective, single-centre, non-randomized cohort study. Inclusion criteria were age > 18 years, hospitalized for an acquired brain injury (ABI), tracheostomized during an ICU stay, and weaned from mechanical ventilation. The exclusion criterion was severe malnutrition. Decannulation failure was defined as recannulation within 96 h after decannulation. Thirty tracheostomized ABI patients from our neurosurgery department were successively and exhaustively included after ICU discharge. Twenty-six patients were decannulated (decannulation rate, 90%). None of them were recannulated (success rate, 100%). Two patients never reached the decannulation stage. Two patients died during the procedure. Mean tracheostomy weaning duration (inclusion to decannulation) was 7.6 (standard deviation [SD]: 4.6) days and mean total tracheostomy time (insertion to decannulation) was 42.5 (SD: 24.8) days. Our results demonstrate that our protocol might be able to determine without instrumental assessment which patient can be successfully decannulated. Therefore, it may be used safely outside ICU or a specialized unit. Moreover, our tracheostomy weaning duration is very short as compared to the current literature.

**Supplementary Information:**

The online version contains supplementary material available at 10.1007/s00455-023-10641-7.

## Introduction

Tracheostomy is a very common ICU procedure. Around 10% of the patients under mechanical ventilation will undergo tracheostomy [1]. In ABI patients, tracheostomy is also frequently performed due to an inability to protect the airway and a poor neurological status incompatible with safe swallowing, as well as to facilitate mechanical ventilation weaning, airway sputum management, and ICU discharge [2]. Tracheostomy tube insertion can be done surgically or using percutaneous technique such as described by Ciagila [3, 4]. Due to the large and quite recent adoption of the percutaneous technique allowing tracheostomy placement in the ICU [5–7], the amount of tracheostomized ABI patients is expected to increase. However, despite its numerous advantages in the ICU, tracheostomy itself may exacerbate dysphagia due to mechanical effects (e.g. reduced upper oesophagus sphincter opening, reduced hyo-laryngeal movements, local laryngeal, pharyngeal and oesophageal lesions, loss of subglottic pressure) and neurophysiological complications (e.g. impairment in swallowing/breathing coordination, laryngeal deafferentation, reduced laryngeal closure) [8–10]. Moreover, the presence of a tracheostomy at discharge from the ICU is problematic in orienting these patients into health facilities adapted to their condition (i.e. rehabilitation centres or secondary care units). The management of tracheostomized patients is considered a cumbersome process and few facilities accept these patients (partly because of a low patients/care givers ratio). In our French regional state (Nouvelle-Aquitaine with 6,010,289 inhabitants in 2019), only 13 secondary care centres are able to manage tracheostomized ABI patients (with only two or three beds per centre available for these type of patients). In addition, tracheostomy weaning in the ICU is well documented [2] but tends to extend the ICU cost and length of stay. These patients are thus frequently discharged from the ICU tracheostomized.

Due to its iatrogenic consequences on swallowing (described above), it is now widely accepted that, as soon as possible, tracheostomy weaning and tracheostomy tube removal must be considered in ABI tracheostomized patients [9]. This weaning improves swallowing and allows the patient to resume physiological breathing, oral nutrition, and phonation, all of which are fundamental needs, and also promotes rehabilitation and awakening [4, 10, 11]. Paradoxically, there are few recommendations for tracheostomy weaning. Existing guidelines are usually for ICU settings or are not specific of the ABI population [2, 12, 13]. The use of tracheostomy management bundle, tracheostomy specialised teams, and tracheostomy weaning or decannulation protocols seems to reduce decannulation failure, time to decannulation, and occurring of adverse events [12]. These protocols are usually based on cuff deflation and tube capping (with speaking valve or a plug) [14]. However, the choice of relevant clinical criteria remains debated, and the use of instrumental assessment is sometimes proposed in order to secure the decision [15].

In this study, we propose the evaluation of a pluridisciplinary protocol based on a standardized reliable, yet simple, clinical assessment adjusted to each patient’s characteristics, with the aim of allowing for safe tracheostomy weaning for all patients. If proven safe and effective, a decannulation decision could be made through the use of our protocol in a secondary care unit, without requiring a systematic instrumental assessment.

## Materials and Methods

This study was a prospective monocentric non-randomized single-arm cohort study (NCT03512054) approved by the ethical committee of our institution (notice number: 17 12 08).

After discharge from neurological or traumatic ICUs, patients were consecutively and exhaustively enrolled in our tracheostomy weaning protocol before the COVID-19 pandemic, between 20/06/2018 and 20/12/2019 from two neurosurgery units in a French university hospital (Bordeaux). All patients were considered stable at admission.

Inclusion criteria were age ≥ 18 years, hospitalized for an ABI, patient tracheostomized during an ICU stay and weaned from mechanical ventilation, written informed consent from the patient or associated legal representative, and the patient affiliated with or benefiting from the French healthcare system.

The exclusion criterion was severe malnutrition defined by the following: body mass index (BMI) < 16 kg/m^2^ or albuminemia < 20 g/L for age < 70 years; BMI < 18 kg/m^2^ or albuminemia < 30 g/L for age > 70 years (standard criteria at protocol’s writing). Severe malnutrition was chosen as the only exclusion criterion due to its association with poorer functional outcome and increased risk for pneumonia and mortality [16].

Tracheostomy weaning was started immediately after discharge from the ICU and inclusion in the study.

Our tracheostomy weaning protocol consisted of a five-step decision-making logigram, created by our pluridisciplinary team including two neurosurgeons, a physical medicine and rehabilitation physician, an intensivist, three physiotherapists, a speech and language therapist, two nurses, and a health manager. This work was informed by a retrospective review of all of the tracheostomized patients managed in our department between 2014 and 2016 and a literature survey (unpublished results): we recorded 29 decannulations without recannulation within a population of 37 ABI tracheostomized patients.

**Fig. 1:**
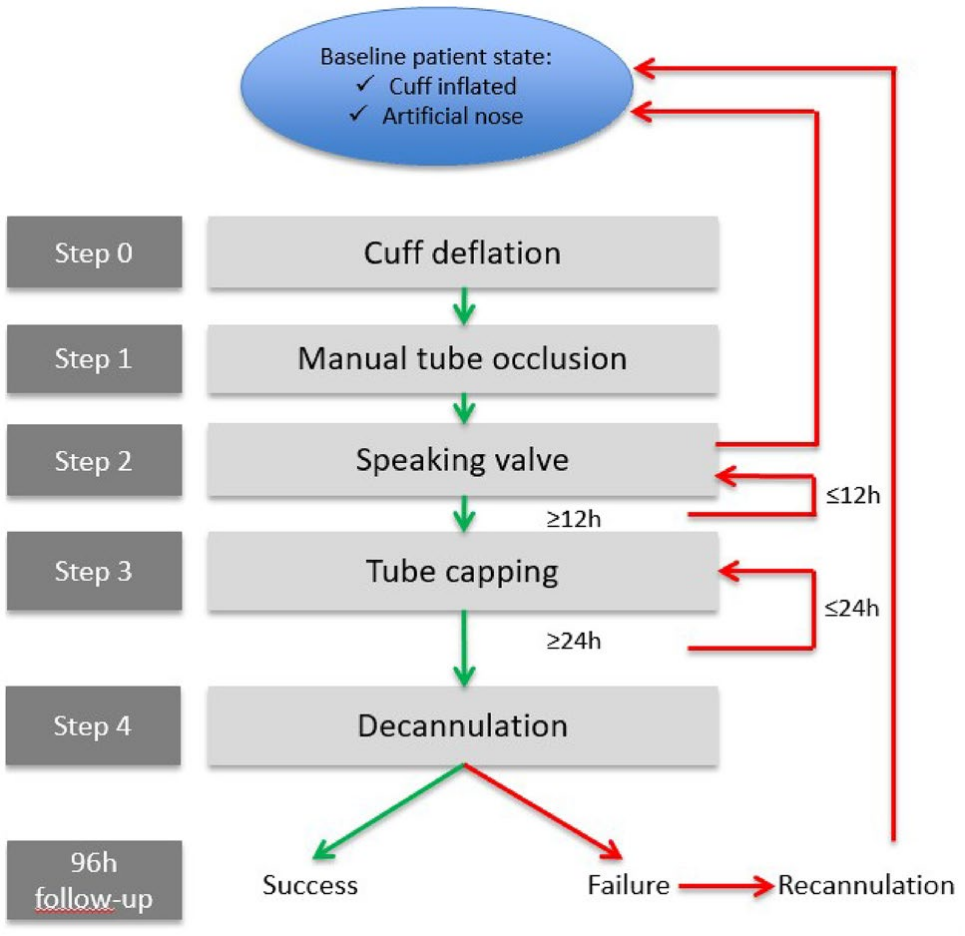
Tracheostomy weaning procedure

The five steps (0–4) consisted of 0/deflating the tracheostomy cuff, 1/manual occlusion of the cannula, 2/placement of a speaking valve for 12 h consecutively, 3/capping the cannula with a plug for 24 h consecutively, and 4/decannulation (final removal of the tracheostomy) (Fig. 1).

Steps 0 and 1 are performed consecutively to assess airway patency. In the case of reduced airway patency or symptoms thereof, tracheostomy downsizing can be performed. Step 2 is designed to promote swallowing rehabilitation [17, 18]. To prevent mucus thickening due to the absence of humidification while breathing through the speaking valve (step 2) [19], this step was reduced to only 12 h. Step 3 promotes swallowing rehabilitation and breathing through the upper airway. Breathing with a plugged tracheostomy (step 3) may increase ventilation workload (mainly because the tracheal lumen is reduced by the presence of the tracheostomy tube) [20]. The 24 h duration was thought to be appropriate, so as to avoid unnecessary prolongation of step 3, while still allowing for the identification of patients with upper-airway obstruction or instability (e.g. obstructive apnoea).

In case of unresolved swallowing or airway patency disorders, an instrumental assessment such as fiberoptic endoscopic evaluation of swallowing (FEES) or video-fluoroscopic swallowing study (VFSS) could have been asked for. But the primary strategy was to manage the patients using only our tracheostomy weaning protocol.

**Fig. 2:**
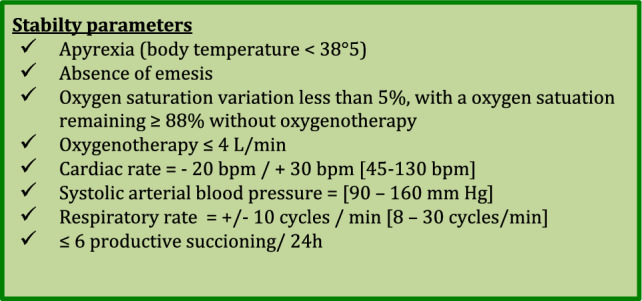
Stability parameters

Our tracheostomy weaning protocol was tailored to each patient, based on the patient’s stability parameters acquired using our monitoring tool. After inclusion, the following vital parameters were recorded with the patient at rest: oxygen saturation, blood pressure, heart rate, respiratory rate, amount of secretion, body temperature, and patient response. Based on these parameters, we created for each patient a bundle of stability parameters, which consisted of the individual parameters, along with a range of minimal and maximal value tolerances (Fig. 2). If the patient fitted the stability parameters, s/he was considered ready to start the weaning protocol. Each step of the protocol was validated, based on the patient’s stability parameters and successful completion of the step within the allotted time duration. If the patient failed to fit the stability parameters, s/he had to go back to the previous step until the stability parameters were met again. With this approach, each patient could go back and forth between steps, depending on his/her own stability parameters, until reaching the final decannulation step. Stability parameters were assessed at each nurse’s visit (at least three times a day) or physiotherapist’s visit (at least once a day). If a decision to go to the next step or to go back to the previous step was taken by the attending caregiver, then the stability parameters had to be assessed within at least 30 min after the step change. Stability parameters were recorded at each assessment.

If a patient was unable to reach the last step during a consecutive 3-month period, then it was determined that this patient was unable to finish the tracheostomy weaning protocol and as such was removed from the protocol (weaning failure). These patients were considered unsuitable for decannulation using our protocol. However, tracheostomy weaning could be continued over the 3-month period using our protocol but without data collection.

To provide maximum autonomy and empowerment regarding the protocol, nurses and paramedical staff were trained to use the tracheostomy protocol (approximately 3 h of courses including stability parameter monitoring, tracheostomy management, swallowing function, and so forth) and to manage intercurrent events that could occur during tracheostomy weaning. After training, a pocket written document containing a brief description of our protocol, the list of the principal intercurrent events, and how to manage them was given to the nurses and care assistants as a reminder (supplemental file 1).

In our protocol, decannulation is a collegial decision based on the stability parameters but as to be validated by the attending medical doctor. The decannulation process was performed by a team comprising a physiotherapist, a nurse, and the attending medical doctor.

Data collection included obtaining information on the decannulation status (failure, success, or never decannulated) and neurological status (at inclusion and unit discharge). Decannulation was considered successful if the patient was still decannulated 96 h after tracheostomy tube removal. Neurological status at inclusion and unit discharge was assessed using the Coma Recovery Scale revised (CRS-r) [21]. Then the patients were classified in terms of the following: 1/unresponsive wakefulness syndrome [CRS-r: 0–7], 2/minimal consciousness state [CRS-r: 8–15], or 3/able to communicate [CRS-r: 16–23]. The CRS-r is widely used to assess disorders of consciousness with ABI patients and is able to detect subtle disorder of consciousness improvements [22]. The severity of swallowing function status at inclusion and unit discharge was described using 7 levels derived from the Dysphagia Outcome Severity Scale (DOSS) (supplemental file 2), assessment was done clinically [23]. Type of lesion (supratentorial, infratentorial, or both) as described in the ICU medical files; reason for tracheostomy placement as described in the ICU medical files. Length of the tracheostomy weaning as the time between inclusion and decannulation; total time of tracheostomy as the time between insertion and decannulation, time between insertion and inclusion, and time between ICU discharge and inclusion in the tracheostomy weaning protocol. Number (total and number per patient) and type of intercurrent events during tracheostomy weaning. Number and type of treatments and associated procedures and their indications (treatment and associated procedures were decided collegially based on the protocol and had to be validated by a medical doctor). FEES during tracheostomy weaning or not. VFSS during tracheostomy weaning or not; presence of ethical limitations or not at inclusion. Type of lesion, type of tracheostomy, feeding status at discharge from ICU, pulmonary status at discharge from ICU, and functional status at discharge from ICU with modified Rankin score (mRS).

All patients also underwent a 6-month follow-up after inclusion to assess long-term tolerance (occurrence of life threatening events or no), vital status, and residency were recorded.

Aside from routine clinical evaluation, tracheostomy management, respiratory therapy, and physical therapy, the patients did not receive any specific evaluation or rehabilitation before and after inclusion.

The study sample size was guided by inclusion feasibility within the two neurosurgery units of Bordeaux University Hospital. The number of eligible patients was estimated retrospectively based on the unit records, and then increased depending on the observed inclusion pace. This was done to improve the analysis precision. Ultimately, we decided to include 30 patients over an 18-month period. Based on the estimated rate of 2–5% of patients unsuitable for decannulation, the predicted number of decannulated patients was 28.

Qualitative data are described in terms of the number of patients, percentage, and 95% confidence interval (CI) according to the exact binomial distribution. Quantitative data are described in terms of number of patients, mean, and standard deviation (SD). Analyses were performed with SAS® software (version 9.4; SAS Institute, Cary, NC, USA).

## Results

After discharge from neurological or traumatic ICUs, 30 tracheostomized ABI patients (12 men) were consecutively and exhaustively enrolled between 20/06/2018 and 20/12/2019 from two neurosurgery units. The mean age was 51.1 (SD: 13.5) years. In all, 15 patients had a supratentorial ABI, 8 patients had an infratentorial ABI, and 7 patients had infratentorial and supratentorial ABI. Mean CRS-r at inclusion was 16.0 (SD: 6.7). The patients were classified as being in the following states: 6 (20%) in an unresponsive wakefulness syndrome, 4 (13%) in a minimal consciousness state, and 20 (67%) as able to communicate. All patients had a 1.0 DOSS score at inclusion.

Reasons for tracheostomy placement were as follows, in order of frequency: impossibility to perform extubation due to neurological status for 14 patients (47%), swallowing disorder for 12 patients (40%), respiratory rehabilitation for 3 patients (10%), and throat inflammatory process for 1 patient (3%). Mean time from tracheostomy to study inclusion was 35 (22.8) days. Mean time from ICU discharge to inclusion was 16.2 (SD: 18.1) days. Mean time for ICU length of stay was 40.2 (16.7) days. Mean time for mechanical ventilation duration was 24.5 (10.7) days.

**Fig. 3:**
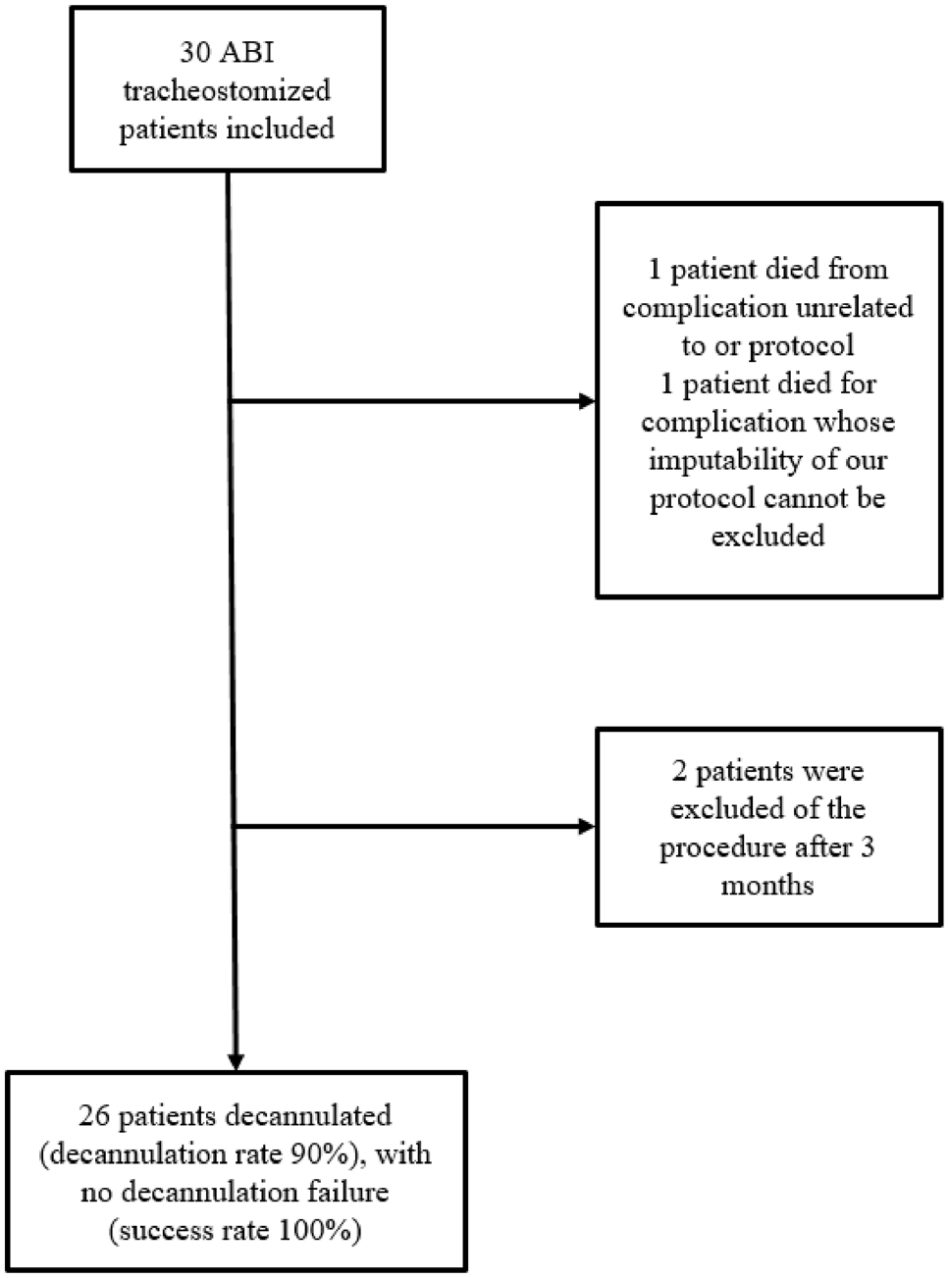
Study flow chart

Of the 30 patients included, 26 were decannulated. All decannulations (100%, 95% CI: 87% to 100%) were successful (see Fig. 3). Two patients were never able to reach the decannulation step after 3 months and were taken off of the weaning procedure according to our protocol (patients 7 and 28), and two patients (7%) died during the weaning period and before decannulation (patients 12 and 13) (Fig. 3). One death was due to pulmonary embolism complications unrelated to our protocol, and the other one was due to cardiorespiratory failure whose imputability to our protocol could not be excluded. Tracheostomy weaning was considered to have failed for this last patient. Thus, our decannulation rate estimated for 29 patients was 90% (95% CI 72, 6–97, 8%). Two auto-decannulations were recorded during tracheostomy weaning. Recannulation was performed in only one patient according to the stability parameters.

The mean duration of tracheostomy weaning (time between the start of tracheostomy weaning and decannulation) was 7.6 (SD: 4.6) days. The mean total tracheostomy time (time between tracheostomy insertion and decannulation) was 42.5 (SD: 24.8) days.

The mean CRS-r at unit discharge was 18.7 (SD: 6.3); 4 patients (15%) were classified as being in an unresponsive wakefulness syndrome, 2 patients (7%) as being in a minimal consciousness state, and 21 patients (78%) as being able to communicate. Mean DOSS at unit discharge was 3.1 (SD: 2.1).

In all, 68 intercurrent events were recorded, with a mean of 2.3 (SD: 2.0) per patient. The four most frequent events were accessory respiratory muscle involvement (21%), anxiety (18%), increase in saliva (10%), and stridor (10%). Complete intercurrent events are described in Table 1. Fifty-two percent of these events were associated with a step-back in tracheostomy weaning and 75% were associated with a treatment or an associated procedure setting. The four main indications for the treatments and associated procedures were accessory respiratory muscle involvement (22%), laboured audible breathing (15%), increase in saliva (12%), and anxiety (10%). Complete indications for treatments and associated procedures are described in Table 2. The four main treatments or associated procedures were aerosol therapy with adrenaline (epinephrine 1 mg) (31%), cannula downsizing (15%), scopoderm patch (scopolamine) (14%), and anxiolytics (10%). Complete treatments and the associated procedure are described in Table 3.

**Table 1:** Complete intercurrent events

Variable	Total		
Intercurrent event	*N*	68	
	Agitation	3	4%
	Anxiety	12	18%
	Excessive bronchial sputum	1	1%
	Saliva disorder	1	1%
	Dyspnoea	1	1%
	Pulmonary embolism	1	1%
	Gastro-oesophageal reflux	2	3%
	Hyperthermia	1	1%
	Hyponatremia	1	1%
	Irritative cough	1	1%
	Respiratory distress	2	3%
	Accessory respiratory muscles involvement	15	21%
	Increased saliva	7	10%
	Sepsis	2	2%
	Laboured audible breathing	7	10%
	Emesis	3	4%
	Pneumopathy	2	3%
	Mucus plug	1	1%
	Thick bronchial mucus	1	1%
	Death due to natural cause	1	1%
	Technical issues	2	3%
	Tracheal oedema	2	3%

**Table 2:** Complete indication for treatment or associated procedures

Variable	Total		
Indication	*N*	58	
	Anxiety	6	10%
	Excessive bronchial sputum	1	2%
	Saliva disorder	1	2%
	Pulmonary embolism	1	2%
	Gastro-oesophageal reflux	2	3%
	Hyperthermia	1	2%
	Hyponatremia	1	2%
	Irritative cough	1	2%
	Oedema	1	2%
	Respiratory distress	2	3%
	Accessory respiratory muscles involvement	13	22%
	Increased saliva	7	12%
	Sepsis	4	7%
	Laboured audible breathing	9	15%
	Pneumopathy	2	3%
	Mucus plug	2	3%
	Thick bronchial mucus	1	2%
	Technical issues	2	3%
	Tracheal oedema	1	2%

**Table 3:** Complete treatments or associated procedures

Variable	Total		
Treatment or associated procedure	*N*	58	
	Aerosol therapy (adrenaline 1 mg)	18	31%
	Scopolamine	8	14%
	Anxiolytics	6	10%
	Cannula downsizing	9	15%
	Antibiotherapy	8	13%
	Treatment for gastro-oesophageal reflux	2	3%
	Treatment for hyponatremia	1	2%
	Treatment for pulmonary embolism	1	2%
	Oxygen therapy	1	2%
	Treatment for sputum excess	2	3%
	One-off tracheostomy weaning interruption	2	3%

At 6 months, 27 patients (90%) were still alive. One of the non-decannulated patients died of a severe digestive complication unrelated to our protocol (patient 7). This death occurred after the patient had been taken off of the weaning procedure 3 months after inclusion, according to our protocol (Table 4). Fifteen patients (55%) were still hospitalized in a secondary care unit [rehabilitation centres (13 patients (50%)), specialized health care facilities (1 patient (4%)), long-term hospitalization centre (1 patient (4%))], 2 patients were still in hospital (7%), 7 patients (26%) were permanently discharged at home, one had day hospitalisation (4%), one had in-house hospitalisation (4%), and one had unknown residency (4%).

**Table 4:** patient’s characteristics

Patient	Decannulation status	CRS-r at inclusion	Ethical restriction at inclusion	FEES during tracheostomy weaning	VFSS during tracheostomy weaning	Vital status during tracheostomy weaning protocol	Etiology severity	Vital status at 6 months	Mechanical ventilation duration (days)	ICU length of stay (days)	Tracheostomy type at ICU discharge	Feeding status at ICU discharge	Pulmonary status at ICU discharge	Functional status at ICU discharge
1	Success	7	Yes	No	No	Alive	SAH (anterior communicating artery aneurysm) W5F4, CVS	Alive	14	40	Portex® 8,5	Gastrostomy, NPO	Eupnea, no O2	mRS 5
2	Success	23	No	No	No	Alive	SAH (left sylvian artery aneurysm) W5F4	Alive	18	26	Portex® 10	Gastrostomy, NPO	Eupnea, no O2	mRS 5, left hemiplegia
3	Success	22	No	No	No	Alive	SAH (left postero inferior cerebellum artery aneurysm) W5F4, CVS	Alive	55	60	Portex® 10	Gastrostomy, NPO	Eupnea, O2 1 l/min	mRS 5
4	Success	21	No	No	No	Alive	Left cerebellar hemangioblastoma	Alive	11	20	Rusch® 10	Gastrostomy, NPO	Eupnea, no O2	mRS 5, left hemiplegia, cerebellar ataxia, left PFP
5	Success	18	No	No	No	Alive	Pituitary macroadenoma	Alive	17	25	Rusch® 10	Gastrostomy, NPO	Eupnea, no O2, history of asthma	mRS 4, left PFP
6	Success	5	No	No	No	Alive	Left temporal ICH	Alive	10	29	Rusch®10	Gastrostomy, NPO	Eupnea, no O2	mRS 5, left hemiplegia
7	Never decannulated	9	Yes	No	No	Alive	Suprasellar cavernoma, post-surgical hemorrhage, right sylvian territory SAH	Deceased (digestive complication, out of protocol death)	31	92	Rusch® 8,5	Gastrostomy, NPO	Eupnea, no O2	mRS 5
8	Success	19	No	No	No	Alive	SAH (anterior communicating artery aneurysm) W4F4	Alive	33	67	Rusch® 8,5	Gastrostomy, NPO	Eupnea, no O2	mRS 5
9	Success	20	No	Yes	No	Alive	SAH (right sylvian artery aneurysm) W5F4,CVS	Alive	33	38	Rusch® 8,5	Gastrostomy, NPO	Eupnea, no O2	mRS 5, left hemiplegia
10	Success	23	No	No	No	Alive	SAH (left internal carotid artery aneurysm), W2F4, CVS	Alive	25	45	Rusch® 8,5	Gastrostomy, NPO	Eupnea, no O2	mRS 4, right hemiplegia
11	Success	5	No	No	No	Alive	SAH (right sylvian artery aneurysm), W5F4	Alive	19	39	Rusch® 8,5	Gastrostomy, NPO	Eupnea, no O2	mRS 5
12	Never decannulated	19	No	No	No	Deceased (pulmonary embolism)	Compressive giant basilar trunk aneurysm	–	20	25	Rusch® 10	Gastrostomy, NPO	Eupnea, no O2	mRS 5
13	Never decannulated	3	Yes	No	No	Deceased (cardio-respiratory failure)	SAH (right sylvian artery aneurysm), W5F4	–	17	47	Rusch® 10	Gastrostomy, NPO	Eupnea, no O2	mRS 5
14	Success	11	No	No	No	Alive	SAH (corpus callosum splenium AVM), W5F4	Alive	17	42	Rusch® 10	Gastrostomy, NPO	Eupnea, no O2	mRS 5
15	Success	17	No	No	No	Alive	Left fronto-temporal ICH + left SDH (left sylvian artery aneurysm)	Alive	24	36	Rusch® 8,5	Gastrostomy, NPO	Eupnea, no O2	mRS 4, right hemiplegia
16	Success	23	No	No	No	Alive	Fourth ventricle hemangioblastoma	Alive	40	43	Rusch® 10	Nasogastric feeding tube, NPO	Eupnea, O2 2 l/min	mRS 4, cerebellar ataxia
17	Success	17	No	No	No	Alive	SAH (basilar trunk aneurysm) W4F4, CVS	Alive	36	41	Rusch® 10	Gastrostomy, NPO	Eupnea, no O2	mRS 5
18	Success	22	No	No	No	Alive	ICH, SAH (left sylvian aneurysm), W4F4	Alive	16	30	Shiley® 6LPC	Gastrostomy, NPO	Eupnea, no O2	mRS 5, right hemiplegia
19	Success	19	No	No	No	Alive	Left pontocerebellar angle meningioma	Alive	27	30	Shiley® 6LPC	Gastrostomy, NPO	Eupnea, O2 3 l/min	mRS 5, right hemiplegia
20	Success	3	No	No	No	Alive	SAH (right internal carotid aneurysm), W5F4, CVS	Alive	27	40	Rusch® 8,5	Gastrostomy, NPO	Eupnea, no O2	mRS 4, left hemiplegia
21	Success	22	No	No	No	Alive	Severe TBI	Alive	38	62	Rusch® 8,5	Gastrostomy, NPO	Eupnea, O2 2 l/min	mRS 5
22	Success	6	Yes	No	No	Alive	SAH (right sylvian aneurysm), W5F4,	Alive	13	20	Rusch® 8,5	Gastrostomy, NPO	Eupnea, no O2	mRS 5, left hemiplegia
23	Success	23	No	No	No	Alive	SAH (anterior communicating artery aneurysm), W4F4	Alive	26	36	Rusch® 8,5	Gastrostomy, NPO	Eupnea, O2 2 l/min	mRS 4, right hemiplegia
24	Success	20	No	No	No	Alive	Left ICH	Alive	38	74	Rusch® 8,5	Gastrostomy, NPO	Eupnea, no O2	mRS 5
25	Success	18	Yes	No	No	Alive	SAH (right sylvian aneurysm), W5F4	Alive	11	38	Rusch® 8,5	Gastrostomy, NPO	Eupnea, no O2	mRS 5, left hemiplegia
26	Success	16	No	No	No	Alive	SAH (right internal carotid aneurysm)	Alive	36	38	Rusch® 8,5	Gastrostomy, NPO	Eupnea, no O2	mRS 5
27	Never decannulated	19	No	No	No	Alive	Posterior fossa astrocytoma	Alive	28	34	Shiley 6LPC	Gastrostomy, NPO	Eupnea, no O2	mRS 5, left hemiplegia
28	Success	14	Yes	No	No	Alive	SAH (pericallosal artery aneurysm), W5F4, CVS	Alive	26	47	Rusch® 8,5	Gastrostomy, NPO	Eupnea, O2 1 l/min	mRS 5
29	Success	12	No	No	No	Alive	SAH (right internal carotid aneurysm), W5F3	Alive	17	22	Rusch® 8,5	Gastrostomy, NPO	Eupnea, no O2, history of OAS	mRS 5, left hemiplegia
30	Success	23	No	No	No	Alive	Superior vermian ICH	Alive	12	21	Rusch® 8,5	Nasogastric feeding tube, NPO	Eupnea, no O2	mRS 4, cerebellar ataxia

*AVM* arteriovenous malformation, *CRS-r* coma recovery scale revised, *CVS* cerebral vasospasm, *FEES* fibreoptic endoscopic evaluation of swallowing, *ICH* intracerebral haemorrhage, *ICU* intensive care unit, *mRS* modified Rankin score, *NPO* nil per os, *PFP* peripheric facial paralysis, *SAH* subarachnoid haemorrhage, *SDH* subdural haemorrhage, *TBI* traumatic brain injury, *VFSS* video-fluoroscopic swallowing studies, W*F* WFNS World Federation of Neurosurgical Societies* Fischer*

Of the 26 patients decannulated with our protocol, none of them were recannulated at 6 months.

## Discussion

The main findings of our study are that we were able to perform decannulation in 86% of the whole population, and 100% of our decannulations were successful. Another important finding is our tracheostomy weaning duration (7.6 [SD: 4.6] days), which appears to be rather short compared to the current literature (19–72 days) [24–27]. Conversely, our total tracheostomy time (42.5 [SD: 24.8] days) appears to be similar to what is commonly reported (25–74 days) [24, 28, 29].

Our results suggest that our protocol may be efficient for assessing which patient is ready to be successfully decannulated, such that most will reach decannulation. Indeed, a high decannulation success rate can be associated with very conservative weaning protocols, thus with a low decannulation rate. However, this was not the case in our study, in which almost all of the participants were decannulated and all of our decannulations were successful. However, such a high success rate could also be interpreted as a consequence of unnecessary tracheostomy and easier tracheostomy weaning as a result. Although, the mean CRS-r at inclusion was 16.0 (SD: 6.7) and one-third of our 30 patients were classified as being in an unresponsive wakefulness syndrome or minimal conscious state at inclusion, almost half of our patients were tracheostomized for “neurological status incompatible with extubation”. In addition, other than having a low level of consciousness, swallowing disorders can lead to tracheostomy weaning failure [11, 15, 30]; in our study, all of our patients had impaired swallowing function at inclusion and almost half of our patients were tracheostomized for a “swallowing disorder”. Notably, all of the patients in our cohort were included exhaustively and consecutively in 2 units that are used to receive all the brain-injured patients at ICU discharge (traumatic and non-traumatic ABI). Our hospital is the largest of our state and the only one receiving severe ABI patients in ICU. This means that almost all tracheostomized ABI patients were discharged from ICU in our 2 neurosurgery units at the time of the study. Thus, we believe that our study population is representative of tracheostomized ABI patients that clinicians are used to managing.

Many of our patients could have started the tracheostomy weaning protocol earlier and even might have been decannulated in the ICU. However, delaying tracheostomy weaning after ICU discharge to the ward was imposed by the study protocol, as we wanted to assess the safety and efficiency of our tracheostomy weaning protocol outside of the ICU. Thus, our patients all benefited from “late” tracheostomy weaning (i.e. in which the mean time between tracheostomy insertion and inclusion was 35.0 [SD: 22.8] days).

Our results then clearly pose the “early vs. late” tracheostomy weaning dilemma. Generally, recent studies tend to advocate for early rehabilitation for ABI patients [31]. Are these concepts necessarily transposable to tracheostomy weaning? The ICU can arguably be considered the safest place to perform tracheostomy weaning. It provides a higher number of caregivers, monitoring facilities, and re-intubation or rescue procedures that can easily be performed by on-the-spot intensivists [2, 14]. Early tracheostomy weaning can therefore be started during the patient’s ICU stay [14, 15]. However, ABI patients’ critical statuses can be an obstacle and a cause of weaning failure in the acute phase [26]. By delaying tracheostomy weaning (i.e. mean delay was 35.0 [22.8] days in our study) at discharge from the ICU, our patients may have been able to attain respiratory, haemodynamic, and more importantly neurological stability. This may explain our very short tracheostomy weaning duration and high success rate. Moreover, before inclusion, all patients received routine physiotherapy and respiratory therapy delivered by the attending ICU physiotherapists. It is possible to consider that it has participated to the general improvement of these patients before inclusion. In a precedent study on a comparable population, early rehabilitation (motor, sensory and sometimes verticalisation) resulted in an earlier decannulation (61 vs 94 days for the delayed rehabilitation group). It has to be noted that our mean total time to decannulation remains shorter [3].

Concerns can be raised that because our protocol started only after ICU discharge, it could have been responsible of prolonged cannulation, which is thought to cause tracheal lesions such as tissue granulation, oedema, and tracheomalacia [32]. However, our total tracheostomy time is similar to what is found in the literature [24, 28, 29]. By delaying tracheostomy weaning, we did not shorten the total tracheotomy time, but tracheostomy weaning may have been easier, safer, and had a high success rate. Moreover, if tracheostomy weaning had been started in the ICU, it may have lengthened the ICU length of stay and increased hospitalization costs, as the ICU cost is higher than the ward cost (in our hospital, the cost of one day in the neurosurgery unit and in the neurological ICU are 655.86€ and 1115.39€, respectively). Our tracheostomy weaning protocol might be safe enough to be performed outside of the ICU without systematic instrumental examination, with a high success rate, and without lengthening of the total tracheostomy time. Thus, tracheostomized ABI patients could be discharged earlier from the ICU, without having completed or even started tracheostomy weaning. Counterintuitively, delayed tracheostomy weaning in ABI patients could be a potential resource that offers cost savings. However, our protocol needs to be tested against a FEES guided one in order to confirm this hypothesis.

One of the specificities of our protocol is the absence of systematic instrumental assessment such as FEES or VFSS, which are considered gold standard evaluations for swallowing disorders [33, 34]. They can be used as an effective tool to guide tracheostomy weaning; FEES, in particular, can be very useful for diagnosing vocal cords impairments or pharyngo-laryngeal lesions such as tissue granulations, all frequently associated with swallowing disorders and thus tracheostomy weaning failure [34–36]. Warnecke et al. have proposed a FEES-guided protocol that seems to be faster, safer, or with less false negatives [15]. Unfortunately, FEES is not always immediately available outside of the ICU or ear, nose, and throat (ENT) ward and depends almost exclusively on medical doctors in our country. VFSS for its part is not a bedside assessment and requires moving the patient to the radiology unit, which is not always appropriate at the acute or sub-acute phase. In our country (France) and in many others, relying on instrumental assessment only to manage all the tracheostomised patients with reasonable delay and to decide whether a patient should be decannulated or not would simply be impossible. Thus it might be responsible of unnecessary prolonged cannulation that are very risky too (38)(Cheung & Napolitano. 2014). One of our goals was to create a tracheostomy weaning protocol that can be used under medical supervision by a nonmedical team and that is based almost solely on clinical examination. Reverberi et al. stated that instrumental assessment is not always available or even feasible with ABI patients, and might be for selected cases only [9]. They suggested that most of the patients should be able to undergo a tracheostomy weaning protocol based only on clinical parameters [9]. Our aim was to create a protocol that is strong enough to minimise the risks (without ignoring them) for these patients and to detect which one really needs instrumental assessment. Thus, we replaced direct objective instrumental evaluation with indirect clinical assessment. For example, swallowing disorders and aspirations were revealed by respiratory signs such as an increase in suctioning and sputum, an increase in the respiratory rate, and/or an increase in the body temperature (as a consequence of lung infection). Default in airway patency (caused by tissue granulation or oedema, or by vocal cord paralysis for example) was revealed by respiratory noises such as a laboured audible breathing particularly if associated with an increased respiratory rate and/or accessory respiratory muscle involvement. Therefore, we did not consider FEES and VFSS as mandatory examinations, and management of intercurrent events was determined based directly on our clinical assessments. For example, cannula downsizing was performed at the first instance, to treat obstruction without a prior FEES. Nevertheless, in our protocol, FEES or VFSS could have been planned for patients with unresolved suspicion of tracheal stenosis or unmanageable swallowing disorders. It happened only for one patient in our cohort during tracheostomy weaning (patient 9 with FEES only). FEES was performed by an ENT resident, as we suspected a tissue granulation after an episode of respiratory distress during the night; it did not reveal tissue granulation, and a mucus plug was suspected. In one study using FEES, it was found out that the presence of tracheal lesions (tissue granulation and oedema were by far the most common) was rarely the cause of decannulation failure [32]. In this study, treatment options were tube change, laser, systemic or nebulized steroid therapy or combined therapy [32]. It is what we proposed in our protocol. The difference is that we would start with steroid therapy or tube change without prior FEES and eventually relied on FEES in case of failure.

We could also have used the blue-dye test to detect silent aspirations. However, despite having an excellent specificity (100%), it is a very low sensitive test (10%) [37].

Swallowing disorders might have been misdiagnosed or underdiagnosed because of the lack of instrumental assessment. Pulmonary infection is one of the major complications of swallowing disorders and can lead to death or delayed discharge [38]. However, in our cohort, we only had 2 pneumonia (3% of the total intercurrent events). They were successfully treated by antibiotherapy (Table 1, 2, 3). A study conducted in a population of subarachnoid haemorrhage (SAH) tracheostomized patients had a post-tracheostomy pneumonia rate over 10% which is fairly higher than ours [29].

The absence of objective assessment could be seen as a limitation of this study, as well as the absence of swallowing rehabilitation. However, we believe that tracheostomy weaning might be seen as a good way to functionally assess swallowing disorders (with cuff deflation and tube capping and a careful monitoring) at least concerning airway protection and non-alimentary swallowing, and also the best way to offer swallowing rehabilitation. We choose not to assess alimentary deglutition during tracheostomy weaning because all our patients had enteral nutrition (mostly gastrostomy). Alimentary-swallowing could be easily tested later, after decannulation. Waiting for the patient to be able to perform alimentary deglutition in order to decannulate might be responsible of unnecessary prolonged cannulation if the patient is already able to manage saliva. Moreover, there are evidences that mild dysphagia is not a strong argument against decannulation in this population (Enrichi et al. 2017). We believe that our protocol is able to detect patients unable to manage saliva and/or with severe dysphagia (as they would not be able to pass through our protocol steps) and thus prevent them to be decannulated.

We did not include cough assessment as a criteria for decannulation. Many protocols suggest that a strong cough might be a good predictor of decannulation readiness [39]. Accuracy of such classification (weak vs strong) remains questionable until you perform cough instrumental assessment. Bach et Saporito have proposed a peak cough flow (PCF) > 160 l/min as a cut-off value [40]. However, the population was composed of neuromuscular patients (e.g. amyotrophic lateral sclerosis). Despite having serious disorders these patients have few cognitive disorders and are usually able to actively participate to such testing. ABI patients are rarely able to do so. To our knowledge, only one study has described an induced peak cough flow (IPCF) suitable for ABI patients [41]. Accuracy, sensitivity, and specificity for successful decannulation were, respectively, 75%, 85,7%, and 54,7% with an optimal cut-off point of 29 l/min [41]. Here again we wanted to create an easy tracheostomy weaning protocol. The IPCF described by Chan et al. needs specific material and can be difficult to perform. Thus we choose not to include a specific cough evaluation.

Concerning the intercurrent events, aside from anxiety, the main two of them (i.e. accessory respiratory muscle involvement and a laboured audible breathing) can be associated with reduced airway patency, which can have multiple causes (e.g. vocal cord paralysis or tissue granulation) [42, 43]. The most frequent treatments and associated procedures (i.e. aerosol therapy with adrenaline and downsizing cannula) performed in our study are directly linked to these main intercurrent events. Moreover, with a mean of 2.3 per patient, the number of intercurrent events was quite low in our study. These events appear to be easily identifiable with clinical assessment, not so frequent, and quite easy to manage. This highlights the fact that tracheostomy weaning of an ABI patient outside of the ICU may not be as overwhelming as once thought.

Interestingly, anxiety, a frequent intercurrent event in our study, was not as frequently treated with medication as accessory respiratory muscle involvement or a laboured audible breathing. In fact, anxiety was probably more of a one-off state for the patients than a general one, and it was likely to be associated with intercurrent events; thus, it does not appear to require long-term drug therapy for many of our patients. A caregiver’s accompaniment or treatment for intercurrent events appears to have been sufficient to reduce anxiety in our patients. However, the use of anxiolytics may be helpful in some cases, particularly when the patient is not able to understand or to participate (e.g. in the case of comprehensive aphasia) [44, 45]. However in our study, anxiety was only clinically assessed by the whole team and decision to treat was taken collegially. This can be a cause of mis-diagnosis. But to our knowledge, there is no anxiety scale available for ABI patients with such disabilities and communication disorders.

Scopolamine has been used successfully to treat excess saliva, usually a sign of a swallowing disorder in which the patient’s swallowing frequency or efficiency is reduced. Because it thickens saliva and reduces its production, scopolamine must be used with caution to avoid mucus plug development. However, its use remains controversial and there is no clear evidence of its efficiency [46, 47].

The main limitations of our study were the relatively small number of patients in our cohort and its monocentric nature. Indeed, our protocol must be tested on a larger scale, in multiple centres and against a FEES-guided protocol. In effect, we believe that our high rate of successful decannulation does not rely only in our team’s pre-existing experience with tracheostomy weaning, or in the skills of a few clinicians. Even if team-based or multidisciplinary tracheostomy weaning had already shown its superiority over standard care ones [48], the additional strength of our protocol seems to lie in its ability to precisely drive caregivers through the use of our logigram, giving them the tools to prevent, recognize, and manage adverse events associated with tracheostomy weaning in ABI patients. Moreover, decannulation can be considered a rather stressful event for caregivers, the patients, and their families. Thus, the framework provided by our procedure secures the weaning and decannulation processes. However, even with larger-scale multicentric studies, it will never be possible to create a protocol capable of preventing every tracheostomy weaning failure. A “zero risk” tracheostomy weaning and decannulation protocol will probably never exist. However, we believe that, with our protocol, the risk of failure can be controlled to the maximum extent and is worth the effort with regard to the benefits of decannulation for these patients. In addition, team education as planned in our protocol is probably one of the keys for implementing a safe and efficient procedure.

Our death rate is 6% (2 patients). It seems to be fairly acceptable considering that tracheostomized ABI patients are usually patients with very severe disabilities and poor outcomes. Moreover, it is quite comparable with the current literature (between 4 and 21% in the decannulation failure group in Küchler et al. [49], between 5 and 6% in Huang et al. [50]). Additionally, the overall mortality in tracheostomized patients has been shown to range from 22 to 45%, which is way over our mortality rate [51].

Of the two patients who died (patients 12 and 13), the question of the imputability of our protocol remains for one (patient 13). According to our protocol and the patient’s stability parameters, tracheostomy weaning had been stopped. Although direct imputability could not have been established, it may be related to the low neurological status of this patient at inclusion (CRS-r = 3). Notably, five patients had a very low CRS-r at inclusion (below 7 and considered to be in an unresponsive wakefulness syndrome) and could have been decannulated (patients 1, 6, 11, 20, 22, see Table 4). In addition, this patient (patient 13) was considered a very severe case, with a poor recovery prognosis. Prior to inclusion, ethical decisions (limitation of active therapeutics) were considered accordingly by the medical staff and the patient’s relatives. However, we decided to include this patient considering the potential benefits of decannulation. Moreover, here again, four patients had the same ethical restriction but were decannulated successfully and were still alive at 6 months (patients 1, 22, 25, and 28, Table 4). Notably, one patient who had ethical restrictions at the inclusion died during the 6-month follow-up (patient 7); however, this patient was not able to reach decannulation and was excluded from the tracheostomy weaning procedure after 3 months, according to our protocol. Evidence of clear predictive factors is still lacking to determine which patients are able to undergo tracheostomy weaning and decannulation. Thus, we chose to include patients regardless of their neurological status. Our protocol was used as a decision-making logigram, giving every patient a chance to reach decannulation.

At 6 months, 55% of our patients were still hospitalized. Considering our national care system and the severity of these patients, our proportion of hospitalized patients is not unusual. To note, some of them are even benefiting of home hospitalisation (1 patient) or day time hospitalisation (1 patient). Moreover, if these patients would not have been decannulated, it is very likely that they would have remained in our unit.

These days, despite the growing research, tracheostomy weaning and particularly decannulation in ABI patients still resemble more of an art form than a well-established science. Due to the lack of evidence, this procedure is usually considered to be unsafe or overwhelming outside of an ICU or specialized unit. In this study, we evaluated the feasibility of such a process using a protocol tailored to patients based on the stability of their condition, allowing every patient to undergo safe, yet efficient, tracheostomy weaning. The decannulation decision was made through the use of our protocol, without the help of systematic instrumental assessment such as FEES or VFSS. Given a decannulation rate of 90% and a success rate of 100% in this study, we believe that our protocol might be used outside of the ICU or specialized unit by a pluridisciplinary non-medical team under medical supervision. The safety and efficiency of the protocol are based on team education and coordination and should be evaluated against instrumental evaluation with a randomized controlled study. Because these are results of a pilot study, we think that we must warn the reader against a misuse of our protocol especially before a controlled study has been performed.

### Supplementary Information

Below is the link to the electronic supplementary material.

Supplementary file1 (DOCX 12 KB)

Supplementary file2 (PDF 217 KB)
