# Supplementary material for: A Pluridisciplinary Tracheostomy Weaning Protocol for Brain-Injured Patients, Outside of the Intensive Care Unit and Without Instrumental Assessment: Results of Pilot Study
Source: Dysphagia. 2023 Dec 7;39(4):608–22. doi: 10.1007/s00455-023-10641-7 (PMC11239749; doi:10.1007/s00455-023-10641-7)
Supplement: Supplementary file 1 — Supplementary file1 (DOCX 12 KB) [file 455_2023_10641_MOESM1_ESM.docx]

**Seven level of swallowing status adapted from the Dysphagia Outcome and Severity Scale (DOSS) O’Neil 1999.**

*Level 1:* Severe dysphagia: NPO: Unable to tolerate any P.O. safely

*Level 2:* Moderately severe dysphagia: Maximum assistance or use of strategies with partial P.O. only (tolerates at least one consistency safely with total use of strategies)

*Level 3:* Moderate dysphagia: Total assist, supervision, or strategies, two or more diet consistencies restricted

*Level 4:* Mild–moderate dysphagia: Intermittent supervision/cueing, one or two consistencies restricted

*Level 5:* Mild dysphagia: Distant supervision, may need one diet consistency restricted

*Level 6:* Within functional limits/modified independence

*Level 7:* Normal in all situations
