# Supplementary material for: A Pluridisciplinary Tracheostomy Weaning Protocol for Brain-Injured Patients, Outside of the Intensive Care Unit and Without Instrumental Assessment: Results of Pilot Study
Source: Dysphagia. 2023 Dec 7;39(4):608–22. doi: 10.1007/s00455-023-10641-7 (PMC11239749; doi:10.1007/s00455-023-10641-7)
Supplement: Supplementary file 2 — Supplementary file2 (PDF 217 KB) [file 455_2023_10641_MOESM2_ESM.pdf]

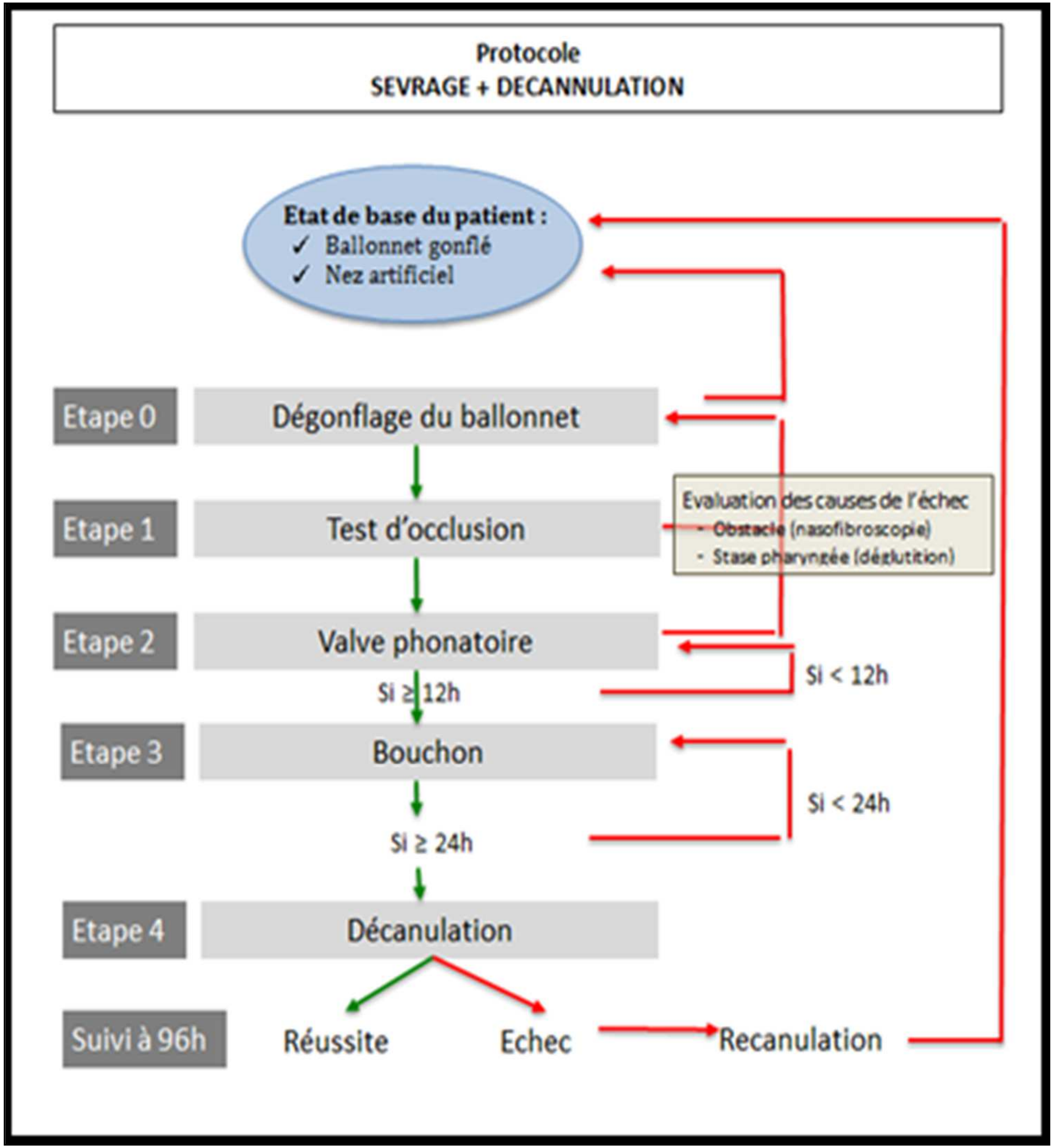

Etude clinique DECATRAC

Code promoteur: CHUBX 2017/31
Promoteur: CHU de Bordeaux

Contacts

Investigateur coordonnateur
Thomas GALLICE – masseur kinésithérapeute référent
Service NEUROCHIRURGIE B
Hôpital Pellegrin - CHU de Bordeaux
☎ : 72 575
thomas.gallice@chu-bordeaux.fr

Co-investigateur
Manuella BALAGUER – masseur kinésithérapeute référent
Service NEUROCHIRURGIE A
Hôpital Pellegrin - CHU de Bordeaux
☎ : 70 035
manuella.balaguer@chu-bordeaux.fr

Infirmiers experts référents
Julie GAUBE ☎ : 95 574
julie.gaube@chu-bordeaux.fr

Clément BARTHELEMY ☎ : 21 211
clement.barthelemy@chu-bordeaux.fr

Service NEUROCHIRURGIE B
Hôpital Pellegrin - CHU de Bordeaux

Attachée de recherche clinique (ARC)
Ouidad HASNAOUI - BEN KACEM
Service NEUROCHIRURGIE B
Hôpital Pellegrin - CHU de Bordeaux
☎ : 21 312 - Fax : 95 984
ouidad.hasnaoui@chu-bordeaux.fr

Conduite à tenir

Lors de la procédure de sevrage, différents événements peuvent se présenter. Voici la liste des principaux événements et la prise en charge à assurer.

Cas n°1 : Hyperthermie > 38,5°

Arrêt du sevrage et voir la conduite à tenir avec le médecin

Cas n°2 : Vomissements ➔ risque important d'inhalation

Arrêt du sevrage : regonfler le ballonnet ET mettre le nez artificiel

Cas n°3 : Cornage ou stridor = bruit anormal lié au rétrécissement des voies aériennes, associé à une détresse respiratoire importante ➔ agir immédiatement

-> 2 cas :

1) Sécrétions « bloquées » en fond de gorge ou sur le pourtour de la canule : utiliser l'aérosol de sérum physiologique et favoriser les efforts de toux ➔ **doit permettre une amélioration immédiate**

2) Autre (ex: granulome, sténose trachéale) : normalement détectés aux étapes 0 - 1 (peu probable lorsque le sevrage en est déjà à l'étape 2 et 3) ➔ **arrêt du sevrage** et réévaluation en équipe pluridisciplinaire de la suite de la prise charge.

Cas n°4 : Dégradation modérée des critères de stabilité à type de désaturation et/ou dégradation de la clinique respiratoire (tirage), augmentation de la fréquence respiratoire, de la fréquence cardiaque ou de la tension artérielle

-> Rechercher une cause

1) Encombrement

- nettoyer la canule (bouchon muqueux)
- Broncho-aspiration (**attention encombrement « silencieux » si antécédents de sécrétions très épaisses et collantes)**

Ces deux manœuvres doivent permettre **une amélioration rapide.**

Dans un second temps : évaluer s'il y a une majoration de l'encombrement (**rappel : maximum 6 aspirations productives** abondantes ou très abondantes **sur 24 heures**).

2) Fatigue respiratoire liée au sevrage

- évaluer un état d'anxiété ou d'agitation qui pourrait influencer négativement la clinique respiratoire
- discuter soit de l'ajout d'oxygène (max 4L), soit du retour à une étape précédente en fonction de la tolérance et des capacités actuelles du patient.

  Doit permettre une **amélioration relativement rapide** (30 minutes max)
- discuter en équipe pluridisciplinaire des causes

3) Dégradation de cause inconnue et autre que celles ci-dessus : **prévenir le médecin et discuter de l'arrêt du sevrage et de la conduite à tenir.**

Cas n°5 : Encombrement important (> 6 aspirations abondantes et très abondantes par 24h)

- Aspirer les sécrétions bronchiques
- Contacter le kinésithérapeute pour kinésithérapie respiratoire
- Effectuer un retour à l'état de base (ballonnet gonflé, nez artificiel)
- Puis discuter en équipe de :

1) Evaluer la qualité et la quantité de l'encombrement pour évaluer la cause

- est-ce des fausses routes à la salive ?
- une pneumopathie en cours d'installation ?

2) Est-ce une mauvaise gestion par le patient de ses sécrétions salivaires (stases buccales, « bavage », …)?

- discuter de la mise en place de Scopoderm®

3) Y-a-t-il des facteurs majorant des troubles de la déglutition qui pourraient être corrigés ?

- reflux gastro-œsophagiens : privilégier la position assise au maximum (**rappel : +30° minimum** pour tout patient trachéotomisé), mise en place de traitement
- sonde nasogastrique : discuter de la mise en place d'une gastrostomie
- mycose buccale : mise en place de traitement

Cas n°6: Auto-décanulation par le patient

1) Mettre le patient en position demi-assise avec le masque à oxygène à 6L
2) Donner l'alerte et faire appeler le médecin et le kiné en urgence
3) Surveiller en continu les constantes vitales, la saturation et la clinique respiratoire
4) En cas de détresse respiratoire aigüe : utiliser l'écarteur de Laborde qui se trouve dans le chariot d'urgence, augmenter le débit d'oxygène
5) Si le patient est stable, attendre l'arrivée du médecin qui doit être très rapide
6) En attendant l'arrivée du médecin, s'assurer qu'une canule de rechange de même modèle et de même diamètre soit prête à l'emploi

Cas n°7 : Décanulation programmée

Prévoir le matériel suivant:

- Masque à oxygène et débitmètre à oxygène
- Chariot d'urgence qui comprend BAVU et pince de Laborde
- Matériel de recanulation avec trachéotomie de même modèle et de même diamètre
